# The digestible indispensable amino acid score (DIAAS) in eggs and egg-containing breakfast meals is greater than in toast breads or hash browns served without eggs

**DOI:** 10.1017/jns.2024.71

**Published:** 2024-11-12

**Authors:** Natalia S. Fanelli, Juliana C. F. R. Martins, Hans H. Stein

**Affiliations:** 1 Division of Nutritional Sciences, University of Illinois, Urbana, IL, USA; 2 Department of Animal Sciences, University of Illinois, Urbana, IL, USA

**Keywords:** Amino acids, DIAAS, Digestibility, Eggs, Protein quality

## Abstract

The objectives of this experiment were to determine the digestible indispensable amino acid score (DIAAS) for eggs cooked in different forms and in traditional egg-bread or egg-hash brown combinations, and to test the hypothesis that DIAAS in eggs is greater than in breads or potatoes. Nine ileal cannulated gilts (average initial body weight: 51.1 ± 6.0 kg) were allotted to a 9 × 6 Youden square design with nine diets and six 7-day periods. Fried egg, boiled egg, scrambled egg, English muffin, Texas toast, and hash brown were included in the experiment. Six diets each contained one source of protein and three diets were combinations of fried eggs and English muffin, boiled eggs and Texas toast, or scrambled egg and hash brown. A nitrogen-free diet was also used and fed to all pigs in one period. The standardised ileal digestibility (SID) of crude protein (CP) and amino acids (AA) was calculated, and DIAAS was calculated for the individual ingredient and combined meals for children between 6 and 36 months and individuals older than 3 years. For both age groups, all cooked eggs had greater (P < 0.001) DIAAS compared with the other foods, and hash brown had greater (P < 0.001) DIAAS than both breads. All combined meals had DIAAS greater than 75 and there were no differences between measured and predicted DIAAS for the combined meals. In conclusion, eggs have ‘excellent’ protein quality for individuals older than 6 months and can compensate for the lower protein quality in plant-based foods, and DIAAS obtained from individual ingredients are additive in mixed meals.

## Introduction

Eggs are high quality proteins that contain all indispensable amino acids (AA) needed for muscle development and body functions. In contrast, most plant proteins have lower protein quality compared with animal-based proteins due to lower concentration and digestibility of AA and the presence of anti-nutritional factors.^(1,2)^ In 2013, the Food and Agricultural Organization (FAO) published a report in which it was recommended that food proteins should be evaluated by using the digestible indispensable amino acid score (DIAAS) method that estimates protein quality by measuring the digestibility of each AA at the end of the small intestine.^(3)^ The reason for this recommendation was that it was concluded that determining protein quality based on total tract digestibility of crude protein (CP) results in an over-estimation of low-quality proteins, whereas high quality proteins are under-valued. In contrast, if protein quality is based on the ileal digestibility of each individual AA, a more accurate assessment of protein quality can be obtained, which is the reason calculation of DIAAS was recommended.^(3)^ In the same report it was also recommended that if ileal AA digestibility cannot be measured in humans, the pig is the best animal model to use and pigs are, therefore, often used to determine DIAAS values.^(3)^

Using published values for AA digestibility in eggs, calculated raw egg has a DIAAS greater than 100 based on the 6-month to 36-month-old reference pattern,^(2)^ but cooking methods (i.e. boiling, or frying) may affect the concentration of AA in eggs.^(4)^ As a consequence, the impact of different cooking procedures on AA digestibility and DIAAS needs to be taken into account to accurately determine protein quality of eggs in the forms they are consumed by humans.

Because eggs are often consumed in combination with other foods, such as breads or potatoes, a person eating eggs will consume AA from eggs in combination with AA from other foods. It is assumed that cooked eggs have a high DIAAS that can compensate for the low protein quality in plant ingredients if consumed together, but data to confirm this hypothesis have not been reported. Therefore, the objectives of this experiment were to determine DIAAS for eggs cooked in different forms and in traditional egg-style combinations with breads or hash brown, and test the hypothesis that DIAAS in eggs is greater than in breads or potatoes and that the high protein quality in eggs can compensate for the low protein quality in plant-based ingredients. The second hypothesis was that based on the standardized ileal digestibility (SID) of AA, DIAAS in combined meals can be predicted from the individual food ingredients.

## Methods

### Ethical approval

The protocol for this experiment was reviewed and approved by the Institutional Animal Care and Use Committee at the University of Illinois prior to initiation of the experiment (#21244). Female pigs that were the offspring of Line 800 boars mated to Camborough females (Pig Improvement Company, Hendersonville, TN) were used.

### Ingredients and experimental diets

Six protein sources were used (Table 1). White large eggs (grade A) from chickens that were kept conventionally in the prime of their life cycle (26–56 weeks of age) were procured, and three different cooking methods (i.e. frying, boiling, or scrambling) were applied. In addition, two sources of breads (i.e. English muffins and white Texas toast) that were produced from barley and wheat flour were purchased frozen from a local provider (Gordon Choice Brand, Champaign, IL, USA) and cubed hash browns that were produced from cooked potatoes were sourced from the same provider. All eggs were prepared until the yolks were firm with a safe minimum internal temperature of 71 °C as measured with a food thermometer.^(5)^ The frying process included coating a non-stick skillet pan with vegetable cooking spray oil, heating the pan over medium-low heat (149 °C), cracking the eggs in the pan, and frying (over hard) for 3–4 min. The same process was used for scrambling the eggs, but three eggs were first beaten in a bowl to a homogenous mixture with no other ingredients added. The mixture was then poured on the heated frying pan and constantly mixed with a rubber spatula to form scrambles. The boiling process included placing the eggs in a pot with water and bringing them to boil over high heat (238 °C). After boiling started, the pot was removed from the heat and a lid was placed on the pot for 10 min to allow a solid yolk to develop. The breads were purchased in sliced form and no cooking procedures were applied to them, but hash browns were baked in a convection oven (Ninja FOODITM XL Pro Air Oven, DT200 Series, Needham, MA, USA) at 204 °C for 10 min to a minimum internal temperature of 74 °C which was measured with a food thermometer (as directed by the manufacturer).

**Table 1.** Analysed composition of food ingredients, as-fed basis

Item, %	Eggs			English muffin	Texas toast	Hash brown
	Fried	Boiled	Scrambled			
Dry matter	30.89	23.85	26.85	57.25	64.61	32.33
Crude protein	15.97	13.27	14.57	8.53	9.38	3.35
Indispensable AA						
Cysteine	0.42	0.36	0.39	0.17	0.19	0.05
Histidine	0.41	0.34	0.37	0.16	0.18	0.05
Isoleucine	0.94	0.79	0.84	0.28	0.32	0.13
Leucine	1.43	1.19	1.27	0.51	0.58	0.20
Lysine	1.27	1.04	1.12	0.19	0.20	0.17
Methionine	0.55	0.47	0.50	0.10	0.11	0.04
Phenylalanine	0.93	0.79	0.83	0.35	0.41	0.13
Threonine	0.78	0.64	0.70	0.21	0.23	0.12
Tryptophan	0.26	0.22	0.24	0.07	0.09	0.03
Tyrosine	0.63	0.49	0.54	0.15	0.21	0.08
Valine	1.09	0.91	0.97	0.31	0.35	0.16
Dispensable AA						
Alanine	0.91	0.76	0.81	0.24	0.26	0.11
Arginine	1.03	0.85	0.92	0.27	0.29	0.15
Aspartic acid	1.63	1.37	1.45	0.33	0.36	0.69
Glutamic acid	1.99	1.73	1.75	2.55	3.02	0.53
Glycine	0.54	0.45	0.48	0.27	0.31	0.10
Proline	0.55	0.46	0.49	0.73	0.87	0.14
Serine	1.08	0.90	0.96	0.33	0.39	0.11

AA, amino acids.

Ten diets were formulated (Tables 2 and 3). Six diets each contained one protein source (i.e. fried egg, boiled egg, scrambled egg, English muffin, Texas toast, or hash brown) as the only source of CP and AA. Three additional diets were prepared by combining fried eggs with English muffin, boiled eggs and Texas toast, or scrambled eggs and hash brown. A weight ratio of 1:1 between eggs and bread or potatoes was used for the combined diets. The last diet was a nitrogen-free diet that was used to measure basal endogenous losses of CP and AA. Premixes with purified ingredients including corn starch, sucrose, canola oil, synthetic cellulose, minerals, and vitamins, were individually prepared for each diet to allow a final CP concentration of 10% (dry matter basis), except for the hash brown and nitrogen-free diets.^(6)^ Vitamins and minerals were included to meet or exceed current requirement estimates for pigs.^(7)^ Diets also contained titanium dioxide as an indigestible marker. All foods were prepared in a manner that humans would consume them, but they were broken up with a food processor (4-Quart Food Processor with LiquiLock Seal System, WFP16SCND; Waring Commercial, Torrington, CT, USA) into smaller pieces (1–2 cm) before being mixed with the premixes to allow proper homogenisation with the marker for digestibility calculation purposes.^(6,8)^ A sample of each ingredient and of each diet was collected at the time of diet mixing for chemical analysis.

**Table 2.** Ingredient composition of experimental diets^*^

Item, %	Eggs			English muffin	Texas toast	Hash brown	Fried egg + English muffin	Boiled egg + Texas toast	Scrambled egg + hash brown	Nitrogen-free
	Fried	Boiled	Scrambled							
Fried egg	44.90	–	–	–	–	–	29.00	–	–	–
Boiled egg	–	48.40	–	–	–	–	–	30.00	–	–
Scrambled egg	–	–	44.90	–	–	–	–	–	31.50	–
English muffin	–	–	–	80.00	–	–	29.00	–	–	–
Texas toast	–	–	–	–	79.00	–	–	30.00	–	–
Hash brown	–	–	–	–	–	79.00	–	–	31.50	–
Corn starch	55.10	51.60	55.10	7.20	7.50	7.50	20.10	18.00	14.90	77.55
Sucrose	10.00	10.00	10.00	7.20	7.50	7.50	10.00	10.00	10.00	10.00
Canola oil	5.00	5.00	5.00	1.00	1.00	1.00	5.00	5.00	5.00	5.00
Cellulose	3.00	3.00	3.00	1.00	1.00	1.00	3.00	3.00	3.00	3.00
Limestone	0.50	0.50	0.50	–	0.40	0.40	0.30	0.40	0.40	0.55
Dicalcium phosphate	1.80	1.80	1.80	1.80	1.80	1.80	1.80	1.80	1.90	2.10
Sodium chloride	0.40	0.40	0.40	0.40	0.40	0.40	0.40	0.40	0.40	0.40
Magnesium oxide	0.10	0.10	0.10	0.10	0.10	0.10	0.10	0.10	0.10	0.10
Potassium carbonate	0.40	0.40	0.40	0.40	0.40	0.40	0.40	0.40	0.40	0.40
Titanium dioxide	0.40	0.40	0.40	0.40	0.40	0.40	0.40	0.40	0.40	0.40
Vitamin mineral premix^†^	0.50	0.50	0.50	0.50	0.50	0.50	0.50	0.50	0.50	0.50

* All diets, except the hash brown and nitrogen-free diets, were formulated to contain 10% crude protein (dry matter basis).

† The vitamin-micromineral premix provided the following quantities of vitamins and micro minerals per kg of complete diet: vitamin A as retinyl acetate, 10,622 IU; vitamin D3 as cholecalciferol, 1,660 IU; vitamin E as DL alpha-tocopheryl acetate, 66 IU; vitamin K as menadione nicotinamide bisulfate, 1.40 mg; thiamin as thiamine mononitrate, 1.08 mg; riboflavin, 6.49 mg; pyridoxine as pyridoxine hydrochloride, 0.98 mg; vitamin B12, 0.03 mg; D-pantothenic acid as D-calcium pantothenate, 23.2 mg; niacin, 43.4 mg; folic acid, 1.56 mg; biotin, 0.44 mg; Cu, 20 mg as copper chloride; Fe, 123 mg as iron sulfate; I, 1.24 mg as ethylenediamine dihydriodide; Mn, 59.4 mg as manganese hydroxychloride; Se, 0.27 mg as sodium selenite and selenium yeast; and Zn, 124.7 mg as zinc hydroxychloride.

**Table 3.** Analysed nutrient composition of experimental diets, as-fed basis

Item, %	Eggs			English muffin	Texas toast	Hash brown	Fried egg + English muffin	Boiled egg + Texas toast	Scrambled egg + hash brown	Nitrogen-free
	Fried	Boiled	Scrambled							
Dry matter	65.52	59.79	63.72	62.94	71.01	45.06	64.84	64.44	54.17	91.31
Crude protein	7.28	6.20	6.50	6.14	7.08	2.58	6.67	6.41	5.25	0.33
Indispensable AA										
Cysteine	0.16	0.17	0.16	0.13	0.15	0.03	0.16	0.17	0.13	0.00
Histidine	0.17	0.16	0.15	0.13	0.14	0.05	0.16	0.16	0.13	0.00
Isoleucine	0.37	0.36	0.35	0.23	0.26	0.08	0.34	0.34	0.29	0.01
Leucine	0.60	0.58	0.55	0.42	0.46	0.10	0.57	0.56	0.45	0.03
Lysine	0.54	0.51	0.50	0.15	0.16	0.12	0.43	0.40	0.40	0.02
Methionine	0.21	0.21	0.20	0.07	0.09	0.02	0.18	0.18	0.16	0.00
Phenylalanine	0.37	0.36	0.34	0.28	0.33	0.09	0.37	0.37	0.29	0.01
Threonine	0.32	0.30	0.29	0.16	0.18	0.06	0.29	0.28	0.25	0.01
Tryptophan	0.10	0.11	0.10	0.06	0.07	0.03	0.09	0.10	0.09	0.02
Tyrosine	0.20	0.19	0.17	0.13	0.16	0.06	0.19	0.18	0.15	0.01
Valine	0.44	0.43	0.40	0.25	0.28	0.11	0.40	0.40	0.34	0.01
Dispensable AA										
Arginine	0.40	0.39	0.36	0.21	0.24	0.11	0.35	0.34	0.30	0.01
Alanine	0.39	0.38	0.36	0.20	0.21	0.06	0.34	0.34	0.29	0.01
Aspartic acid	0.68	0.66	0.62	0.26	0.28	0.55	0.58	0.56	0.65	0.02
Glutamic acid	0.92	0.88	0.84	2.00	2.37	0.39	1.34	1.46	0.76	0.02
Glycine	0.23	0.22	0.21	0.22	0.25	0.05	0.24	0.24	0.18	0.01
Proline	0.24	0.23	0.22	0.59	0.71	0.06	0.38	0.41	0.20	0.03
Serine	0.45	0.42	0.41	0.25	0.28	0.06	0.40	0.39	0.32	0.01

AA, amino acids.

### Experimental design and digestibility trial

Nine growing prepubertal female pigs (initial body weight: 51.1 ± 6.0 kg) that had a T-cannula installed in the distal ileum^(9)^ were used. Pigs were cannulated when they had a body weight of approximately 25 kg and had been used in a previous experiment and subsequently fed a common grower diet for seven days before being used in the current experiment. Pigs were randomly allotted to a 9 × 6 Youden square design with nine diets and six 7-day periods. During the experiment, no pig received the same diet more than once. There were, therefore, 6 replicate pigs per treatment. The nitrogen-free diet was fed to all pigs in the middle of the experiment (i.e. after the third week) to allow calculation of SID of CP and AA for each pig.^(6)^ Pigs were housed in an environmentally controlled room in individual pens (1.5 × 2.5 m) with smooth sides and half slatted concrete floors. Pens were equipped with a self-feeder and a nipple drinker.

All pigs were fed their assigned diets in a daily amount equivalent to 8% of the metabolic body weight calculated on a dry matter basis. The daily feed allowance was divided into two equal meals that were provided every day at 0700 and 1600 h.^(6)^ Pigs were weighed at the end of each experimental period to calculate the daily feed allowance for the following period. The initial 5 days of each experimental period were considered an adaptation period to the diet. Ileal digesta were collected for 9 hours on days 6 and 7 using standard operating procedures.^(8,9)^ At the end of the experiment, pigs had an average body weight of 75.9 ± 13.3 kg.

### Chemical analyses

At the conclusion of the experiment, ileal digesta samples were thawed, homogenised for each animal and diet, and a sub-sample was collected and lyophilised. Ileal digesta samples were then ground using a coffee grinder prior to chemical analysis. Ingredients, diets, and ileal digesta were analysed for dry matter (Method 930.15)^(10)^ and for nitrogen by combustion (Method 990.03)^(10)^ using a LECO FP628 Nitrogen analyser (LECO Corp., Saint Joseph, MI, USA). Crude protein was calculated as nitrogen × 6.25. Samples of ingredients, diets and ileal digesta, were analysed for AA [Method 982.30 E (a, b, c)]^(10)^ on a Hitachi Amino Acid Analyser (Model L8800, Hitachi High Technologies America Inc., Pleasanton, CA, USA). Diets and ileal digesta samples were also analysed for titanium.^(11)^

### Calculations

Values for apparent ileal digestibility (AID), basal endogenous losses, and SID of CP and AA in the diets were calculated according to published procedures.^(12)^

The predicted AID of AA in the combined diet containing each egg and plant-based protein was calculated using the following equation^(13)^:

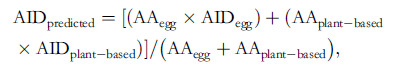

where AID_predicted_ (%) is the predicted AID for an AA in the mixed diet; AA_egg_ and AA_plant-based_ are the concentrations (%) of that AA contributed by each egg and plant-based protein, respectively, which were calculated by multiplying the concentration of that AA (%) in the ingredient by the proportion (%) of the ingredient in the mixed diet; AID_egg_ and AID_plant-based_ are the determined AID (%) of the AA in each egg and plant-based protein, respectively. The predicted AID of CP and the SID of CP and all AA in the combined diets containing each egg and plant-based protein were calculated using the same equation.

The digestible indispensable amino acid (DIAA) reference ratios were calculated for all proteins and combined meals using the following equation^(3)^:

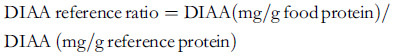




Separate ratios were calculated using the reference protein for two different age groups: child (from 6 to 36 months old) and older child, adolescent, and adult (individuals older than 3 years). The DIAAS for each protein and combined meals were calculated using the following equation^(3)^:

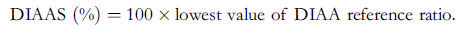




### Statistical analyses

Data were analysed using the MIXED procedure of SAS (SAS Inst. Inc., Cary, NC, USA) using pig as the experimental unit. Normality of residuals and homogeneity of variances were confirmed using the UNIVARIATE procedure. The Brown and Forsythe’s test was also used to check for variance homogeneity, and when this assumption was not met, data was transformed using the BOXCOX procedure and assumptions were re-checked. Outliers were detected as observations that deviated from the treatment mean by ± 3 times the interquartile range. The statistical model included diet as the fixed effect and pig and period as random effects. The LSMEANS were calculated with corresponding standard errors (SE). Within each of the three combined meals, the paired *t*-test was used to test the null hypothesis that the difference between the measured and predicted AID or SID of CP and AA, as well as values for DIAAS for the mixed diets, was not different than 0. Significance was considered at P < 0.05 and tendencies at 0.05 ≤ P < 0.10.

## Results

All pigs remained healthy throughout the experiment and readily consumed their diets.

### Digestibility

The AID of CP and most AA was greater (P < 0.01) in the three cooked eggs and in the two types of bread compared with hash brown (Table 4), but the two breads had lower (P < 0.01) AID of lysine when compared with hash brown and the cooked eggs. The SID of tryptophan was greater (P = 0.008) in hash browns than in all sources of eggs. The SID of lysine was greater (P = 0.03) in the three sources of eggs than in English muffin, and the SID of lysine in scrambled eggs was also greater (P = 0.03) than in Texas toast. The SID of methionine was greater (P = 0.013) in the Texas toast and scrambled eggs than in fried eggs, English muffin, or hash browns. Likewise, the SID of phenylalanine was greater (P = 0.039) in the Texas toast than in fried egg, boiled egg, and hash brown. The SID of tyrosine was lower (P = 0.021) in hash brown compared with fried egg, scrambled egg, and the two breads. There was a tendency for the SID of histidine to be greater (0.05 ≤ P < 0.10) in the Texas toast and hash brown compared with the cooked eggs. No differences were observed among food ingredients for the SID of CP, isoleucine, leucine, threonine, valine, and cysteine.

**Table 4.** Apparent ileal digestibility (AID) and standardized ileal digestibility (SID) of crude protein (CP) and amino acids (AA) in food ingredients^*^

	AID								SID							
Item, %	Eggs			English muffin	Texas toast	Hash brown	SE^3^	P-value	Eggs			English muffin	Texas toast	Hash brown	SE	P-value
	Fried	Boiled	Scrambled						Fried	Boiled	Scrambled					
Crude protein	50.5^a^	51.2^a^	54.8^a^	49.8^a^	55.2^a^	17.9^b^	4.73	<0.001	95.1	97.0	101.3	96.1	101.8	99.6	4.41	0.617
Cysteine	67.3^a^	67.3^a^	71.3^a^	69.5^a^	68.6^a^	–4.1^b^	3.57	<0.001	86.4	85.0	89.7	92.7	92.0	73.7	4.76	0.460
Histidine	68.3^a^	67.0^a^	73.7^a^	69.6^a^	72.2^a^	48.6^b^	2.47	<0.001	87.7	85.4	93.0	92.8	96.6	96.1	3.37	0.057
Isoleucine	78.2^ab^	79.2^ab^	81.9^a^	70.6^c^	75.7^bc^	45.1^d^	1.98	<0.001	91.0	91.5	95.3	90.9	96.3	88.6	2.22	0.112
Leucine	78.6^ab^	79.3^ab^	81.6^a^	75.1^b^	78.7^ab^	36.5^c^	1.82	<0.001	92.1	92.4	96.2	93.8	97.9	91.9	2.71	0.331
Lysine	73.5^a^	73.3^a^	76.6^a^	14.5^c^	19.6^c^	38.4^b^	5.80	<0.001	87.0^ab^	87.2^ab^	91.8^a^	62.5^c^	69.8^bc^	80.5^abc^	5.84	0.030
Methionine	86.2^b^	86.9^b^	90.5^a^	73.3^d^	80.3^c^	43.2^e^	1.92	<0.001	92.1^b^	92.6^ab^	96.8^a^	90.7^b^	96.6^a^	90.1^b^	1.53	0.013
Phenylalanine	78.8^a^	80.4^a^	83.1^a^	78.5^a^	82.0^a^	55.9^b^	1.69	<0.001	91.5^c^	92.4^bc^	96.9^ab^	94.2^abc^	97.7^a^	90.5^c^	1.98	0.039
Threonine	64.4^a^	62.6^a^	68.0^a^	42.5^b^	42.1^b^	4.1^c^	3.50	<0.001	89.4	87.0	93.3	88.0	91.4	90.3	4.62	0.816
Tryptophan	78.5^a^	76.0^a^	78.1^a^	76.0^a^	76.9^a^	62.1^b^	2.18	<0.001	92.2^cd^	89.6^d^	92.7^bcd^	98.9^abc^	99.5^ab^	101.1^a^	2.64	0.008
Valine	74.1^ab^	75.1^ab^	77.1^a^	63.6^c^	69.0^bc^	48.6^d^	2.37	<0.001	90.7	91.4	95.5	92.1	98.3	96.8	3.24	0.218
Tyrosine	73.5^a^	73.0^a^	74.0^a^	65.1^b^	73.2^a^	46.5^c^	2.38	<0.001	90.7^a^	89.9^ab^	93.6^a^	90.0^a^	95.5^a^	82.9^b^	2.38	0.021

SE, standard error.

a–e Mean values within a row with unlike superscript letters are significantly different (P < 0.05).

* Data are means of 6 observations per treatment, except for the hash brown that had 5 observations per treatment. The SID values were calculated by correcting values for basal ileal endogenous losses for each pig as its own control.

The measured and predicted AID values for the combined meal of fried eggs and English muffin differed (P = 0.022) from zero for tryptophan and tended to differ (0.05 ≤ P < 0.10) from zero for histidine, isoleucine, methionine, and cysteine, but for SID, differences tended to differ (0.05 ≤ P < 0.10) from zero only for tryptophan (Table 5). For the combined meal of boiled egg and Texas toast, no differences were observed between the measured and predicted AID or SID values (Table 6). For the combined meal of scrambled egg and hash brown, the measured and predicted AID values differed from zero for leucine (P = 0.044), methionine (P = 0.035), and threonine (P = 0.041), and tended to differ (0.05 ≤ P < 0.10) from zero for cysteine, but for SID, no differences were observed between the measured and predicted values for this meal (Table 7).

**Table 5.** Measured and predicted values for apparent ileal digestibility (AID) and standardized ileal digestibility (SID) of crude protein (CP) and amino acids (AA) in the fried egg and English muffin combined meal^‡,^^§^

	AID				SID			
Item, %	Measured	Predicted	Difference	SE	Measured	Predicted	Difference	SE
Crude protein	44.4	50.3	–5.9	4.66	87.3	95.4	–8.1	4.26
Cysteine	62.8	67.9	–5.1†	2.08	82.3	88.2	–5.8	3.76
Histidine	64.2	68.7	–4.4†	2.01	83.4	89.1	–5.7	3.84
Isoleucine	72.5	76.5	–4.0†	1.89	86.3	90.9	–4.6	2.87
Leucine	74.6	77.7	–3.1	1.72	88.9	92.6	–3.7	3.01
Lysine	59.6	65.6	–6.0	3.32	76.8	83.8	–6.9	4.73
Methionine	81.7	84.1	–2.5†	1.16	88.8	91.9	–3.1	1.62
Phenylalanine	76.2	78.7	–2.5	1.76	88.9	92.2	–3.4	2.89
Threonine	55.8	59.9	–4.1	2.78	83.3	89.1	–5.8	5.33
Tryptophan	70.9	77.9	–7.0*	2.13	87.1	93.7	–6.6†	3.04
Valine	67.9	71.8	–3.9	2.17	86.3	91.1	–4.7	3.59
Tyrosine	68.8	71.9	–3.1	2.53	86.3	90.5	–4.4	3.60

SE, standard error.

‡ Means in a row differ if *Measured vs. predicted P < 0.05, **Measured vs. predicted P < 0.01, or tend to differ if Measured vs. predicted 0.05 ≤ P < 0.10.

§ Data are means of 6 observations per treatment.

**Table 6.** Measured and predicted values for apparent ileal digestibility (AID) and standardized ileal digestibility (SID) of crude protein (CP) and amino acids (AA) in the boiled egg and Texas toast combined meal^‡,^^§^

	AID				SID			
Item, %	Measured	Predicted	Difference	SE	Measured	Predicted	Difference	SE
Crude protein	51.2	52.9	–1.7	4.25	95.6	99.0	–3.4	5.57
Cysteine	69.0	67.8	1.3	3.97	85.9	87.5	–1.6	3.92
Histidine	70.2	68.8	1.5	3.20	87.1	89.3	–2.2	3.18
Isoleucine	79.8	78.2	1.6	2.24	92.3	92.9	–0.6	2.16
Leucine	80.9	79.1	1.9	2.09	93.6	94.2	0.6	2.05
Lysine	66.2	64.6	1.5	5.04	81.8	84.4	–2.5	4.99
Methionine	88.2	85.7	2.6	1.40	94.6	93.3	1.3	1.18
Phenylalanine	83.0	80.9	2.0	2.16	93.9	94.2	–0.3	2.06
Threonine	62.2	57.2	5.0	5.02	87.4	88.2	–0.8	4.95
Tryptophan	76.8	76.2	0.6	3.47	90.8	92.5	–1.7	3.79
Valine	76.7	73.4	3.3	1.75	93.0	93.3	–0.3	1.90
Tyrosine	73.3	73.0	0.2	3.23	89.9	91.6	–1.7	3.15

SE, standard error.

‡ Means in a row differ if *Measured vs. predicted P < 0.05, **Measured vs. predicted P < 0.01, or tend to differ if †Measured vs. predicted 0.05 ≤ P < 0.10.

§ Data are means of 6 observations per treatment.

**Table 7.** Measured and predicted values for apparent ileal digestibility (AID) and standardized ileal digestibility (SID) of crude protein (CP) and amino acids (AA) in the scrambled egg and hash brown combined meal^‡,^^§^

	AID				SID			
Item, %	Measured	Predicted	Difference	SE	Measured	Predicted	Difference	SE
Crude protein	50.0	47.9	2.1	3.90	99.3	101.0	–1.8	3.31
Cysteine	67.1	63.5	3.6^†^	1.67	86.8	88.1	–1.3	2.48
Histidine	69.5	70.4	–0.9	1.82	89.9	93.4	–3.5	1.89
Isoleucine	78.6	77.1	1.5	1.20	92.5	94.4	–1.9	1.21
Leucine	78.8	75.5	3.3*	1.25	94.0	95.6	–1.7	1.55
Lysine	73.3	71.5	1.7	3.47	88.4	90.3	–2.0	3.68
Methionine	89.1	87.1	2.1*	0.74	95.6	96.3	–0.7	0.60
Phenylalanine	81.4	79.3	2.1	1.17	94.9	96.0	–1.1	1.37
Threonine	63.9	58.6	5.3*	1.94	91.3	92.9	–1.6	2.31
Tryptophan	78.0	76.4	1.6	1.63	92.6	93.7	–1.0	1.50
Valine	74.2	73.1	1.1	1.26	92.7	95.7	–3.0	1.61
Tyrosine	71.5	70.3	1.2	1.72	90.0	92.1	–1.9	1.89

SE, standard error.

‡ Means in a row differ if *Measured vs. predicted P < 0.05, **Measured vs. predicted P < 0.01, or tend to differ if^†^Measured vs. predicted 0.05 ≤ P < 0.10.

§ Data are means of 6 observations per treatment.

### Protein quality

For both age groups (i.e., children from 6 to 36 months and individuals older than three years), all three cooked eggs had greater (P < 0.01) DIAAS compared with the other food ingredients, and hash brown had greater (P < 0.01) DIAAS than English muffin and Texas toast (Table 8). There was no limiting AA (DIAAS ≥ 100) for the cooked eggs, but lysine was the first limiting AA in both breads and in hash brown.

**Table 8.** Digestible indispensable amino acids (DIAA) reference ratio and digestible indispensable amino acid score (DIAAS) in food ingredients^*^

Item	Eggs			English muffin	Texas toast	Hash brown	SEM	P-value
	Fried	Boiled	Scrambled					
Child^†^								
DIAA reference ratio								
Histidine	1.14	1.10	1.18	0.87	0.93	0.79		
Isoleucine	1.67	1.69	1.71	0.93	1.03	1.05		
Leucine	1.25	1.25	1.27	0.85	0.92	0.83		
Lysine	1.21	1.20	1.25	0.25	0.26	0.73		
SAA	2.02	2.07	2.14	1.08	1.12	0.76		
AAA	1.72	1.70	1.73	1.07	1.22	1.09		
Threonine	1.41	1.35	1.44	0.68	0.73	1.05		
Tryptophan	1.76	1.75	1.77	1.01	1.13	1.03		
Valine	1.44	1.45	1.48	0.79	0.85	1.07		
DIAAS, %	114^a^	110^a^	118^a^	25^c^ (Lysine)	26^c^ (Lysine)	73^b^ (Lysine)	3.60	<0.001
Older child, adolescent, adult^‡^								
DIAA reference ratio								
Histidine	1.42	1.38	1.48	1.09	1.16	0.98		
Isoleucine	1.78	1.80	1.83	1.00	1.10	1.12		
Leucine	1.35	1.35	1.37	0.92	0.99	0.90		
Lysine	1.44	1.42	1.48	0.30	0.31	0.86		
SAA	2.37	2.43	2.52	1.26	1.32	0.89		
AAA	2.18	2.15	2.20	1.35	1.55	1.38		
Threonine	1.75	1.67	1.79	0.85	0.90	1.30		
Tryptophan	2.27	2.26	2.28	1.31	1.45	1.33		
Valine	1.55	1.56	1.59	0.85	0.91	1.15		
DIAAS, %	135^a^	135^a^	137^a^	30^c^ (Lysine)	31^c^ (Lysine)	86^b^ (Lysine)	3.67	<0.001

AAA, aromatic amino acids (phenylalanine + tyrosine); SAA, sulfur amino acids (methionine + cystein); SEM, standard error of the mean.

a–c Mean values within a row with unlike superscript letters are significantly different (P < 0.05).

* First limiting AA in parenthesis.

† The DIAA reference ratios and DIAAS values were calculated using the recommended AA scoring pattern for a child (6 months to 3 years). The DIAA reference patterns are expressed as mg AA/g protein: His, 20; Ile, 32; Leu, 66; Lys, 57; SAA, 27; AAA, 52; Thr, 31; Trp, 8.5; Val, 43.^(3)^

‡ The DIAA reference ratios and DIAAS values were calculated using the recommended AA scoring pattern for an older child, adolescent, and adult (older than 3 years). The DIAA reference patterns are expressed as mg AA/g protein: His, 16; Ile, 30; Leu, 61; Lys, 48; SAA, 23; AAA, 41; Thr, 25; Trp, 6.6; Val, 40.^(3)^

The measured and predicted DIAA reference ratios for the combined meal of fried egg and English muffin tended to differ from zero (0.05 ≤ P < 0.10) for tryptophan for both age groups (Table 9). However, for both age groups, there were no differences between the measured and predicted DIAA reference ratios for the combined meals of boiled eggs and Texas toast or for scrambled eggs and hash browns. In addition, no differences were observed between the measured and predicted DIAAS regardless of type of meal and age group. Lysine was the first limiting AA for the egg-bread combinations, but there was no limiting AA (DIAAS ≥ 100) for the egg-hash brown combination.

**Table 9.** Measured and predicted values for digestible indispensable amino acids (DIAA) reference ratio and digestible indispensable amino acid score (DIAAS) in combined meals of eggs and plant-based proteins^‡^,^§^

Item	Fried egg + English muffin				Boiled egg + Texas toast				Scrambled egg + hash brown			
	Measured	Predicted	Difference	SE	Measured	Predicted	Difference	SE	Measured	Predicted	Difference	SE
Child^||^												
DIAA reference ratio												
Histidine	0.97	1.04	–0.07	0.05	1.02	1.04	–0.02	0.04	1.04	1.11	–0.04	0.02
Isoleucine	1.34	1.41	–0.07	0.04	1.42	1.43	–0.01	0.03	1.55	1.59	–0.03	0.02
Leucine	1.06	1.11	–0.04	0.04	1.12	1.12	0.00	0.02	1.16	1.18	–0.02	0.02
Lysine	0.80	0.88	–0.07	0.05	0.79	0.81	–0.02	0.05	1.12	1.14	–0.02	0.05
SAA	1.60	1.69	–0.08	0.05	1.69	1.69	0.00	0.05	1.86	1.88	–0.02	0.03
AAA	1.43	1.49	–0.06	0.05	1.50	1.51	–0.01	0.04	1.58	1.61	–0.02	0.03
Threonine	1.08	1.15	–0.07	0.07	1.11	1.11	0.00	0.06	1.33	1.36	–0.02	0.03
Tryptophan	1.39	1.50	–0.11^†^	0.05	1.49	1.51	–0.02	0.06	1.60	1.63	–0.02	0.03
Valine	1.15	1.21	–0.06	0.05	1.22	1.22	0.00	0.02	1.34	1.39	–0.04	0.02
DIAAS, %	80 (Lysine)	88 (Lysine)	–7.28	4.96	79 (Lysine)	81 (Lysine)	–2.43	4.81	104	111	–7.27	3.88
Older child, adolescent, adult^¶^												
DIAA reference ratio												
Histidine	1.21	1.30	–0.08	0.06	1.28	1.30	–0.03	0.05	1.33	1.39	–0.05	0.03
Isoleucine	1.43	1.51	–0.08	0.05	1.52	1.52	0.00	0.04	1.65	1.69	–0.04	0.02
Leucine	1.15	1.20	–0.05	0.04	1.21	1.22	0.00	0.03	1.23	1.28	–0.02	0.02
Lysine	0.95	1.04	–0.09	0.06	0.94	0.97	–0.03	0.06	1.33	1.36	–0.03	0.06
SAA	1.88	1.98	–0.10	0.06	1.98	1.98	0.00	0.05	2.18	2.21	–0.02	0.03
AAA	1.81	1.89	–0.08	0.07	1.90	1.91	–0.01	0.05	2.01	2.04	–0.03	0.03
Threonine	1.33	1.43	–0.09	0.09	1.37	1.37	0.00	0.08	1.64	1.69	–0.03	0.04
Tryptophan	1.79	1.93	–0.14^†^	0.06	1.92	1.94	–0.03	0.08	2.06	2.10	–0.02	0.03
Valine	1.24	1.30	–0.07	0.05	1.32	1.31	0.00	0.03	1.45	1.50	–0.05	0.03
DIAAS, %	95 (Lysine)	104	–8.65	5.89	94 (Lysine)	97 (Lysine)	–2.86	5.71	123	128	–4.58	3.64

AAA, aromatic amino acids (phenylalanine + tyrosine); SAA, sulfur amino acids (methionine + cystein); SE, standard error.

‡ Means in a row differ if *Measured vs. predicted P < 0.05, **Measured vs. predicted P < 0.01, or tend to differ if

† Measured vs. predicted 0.05 ≤ P < 0.10.

§ First limiting AA in parenthesis.

|| The DIAA reference ratios and DIAAS values were calculated using the recommended AA scoring pattern for a child (6 months to 3 years). The DIAA reference patterns are expressed as mg AA/g protein: His, 20; Ile, 32; Leu, 66; Lys, 57; SAA, 27; AAA, 52; Thr, 31; Trp, 8.5; Val, 43.^(3)^

¶ The DIAA reference ratios and DIAAS values were calculated using the recommended AA scoring pattern for an older child, adolescent, and adult (older than 3 years). The DIAA reference patterns are expressed as mg AA/g protein: His, 16; Ile, 30; Leu, 61; Lys, 48; SAA, 23; AAA, 41; Thr, 25; Trp, 6.6; Val, 40.^(3)^

## Discussion

Pigs were used as a model for humans to estimate AA digestibility in this experiment as has been recommended.^(3)^ The reason for this recommendation is that pigs easily tolerate installment of a cannula in the distal ileum, which allows for collection of undigested food materials at the end of the small intestine and before hindgut microbes get access to the undigested AA and potentially ferment them. Use of pigs in AA digestibility experiments, therefore, allow for determining digestibility of AA in multiple food proteins in the same animal, which reduces experimental variation. Comparison of digestibility values between pigs and humans has demonstrated excellent agreement between the two species.^(14,15)^ Indeed, when the ileal AA digestibility of seven food proteins were determined in both pigs and humans, a significant difference was obtained only for total lysine, but not for reactive lysine, thus demonstrating the accuracy of using the pig as a model for humans to determine AA digestibility.^(15)^

Diet analysis indicated that the intended concentration of CP and AA were present in all diets. Likewise, nutrient composition of food ingredients were in agreement with published values.^(16)^ The two breads were produced from wheat and barley flour and SID of some AA were in agreement with published values,^(17–19)^ but with the exception of lysine, SID of most AA was greater in both breads than that reported for wheat or barley,^(20)^ and it is likely that processing positively impacted digestibility of AA as has been demonstrated for other ingredients.^(19)^ However, because lysine was low compared with other AA, heat damage during processing may have occurred in these food products, which has also been previously demonstrated for other food ingredients.^(21,22)^

The SID of AA in hash brown was generally in agreement with published values for potato protein concentrate,^(7)^ but with lower SID of lysine due to possible heat damage during preparation.^(23)^ The SID of AA in the cooked eggs were also in agreement with published values for boiled whole eggs.^(24,25)^ However, available values for digestibility of AA in eggs are typically obtained from ingredients that have previously been rejected for human consumption, and although values for human-grade boiled egg exist, to our knowledge, this is the first time the digestibility of AA is measured in eggs prepared with different cooking procedures. Values for SID in all sources of cooked eggs demonstrated that they have high digestibility as previously demonstrated for several animal-based proteins.^(7,16,26,27)^

The DIAAS for eggs were in agreement with calculated values.^(3)^ However, the DIAAS for both English muffin and Texas toast were lower than values for wheat flour obtained in *in vivo* studies,^(17,18)^ which likely is a result of the heat damage during preparation of these foods that reduced the digestibility of lysine. Corn flakes and Quick Oats also have lower DIAAS than the DIAAS in corn flour, oat flour, and oat protein, respectively.^(22,28)^ These observations clearly demonstrate that processes used to prepare foods for human consumption may result in heat damage and a subsequent reduction in DIAAS. This is particularly a problem for foods based on cereal grains where lysine is the first limiting AA because lysine is the AA that is most negatively affected by heat damage due to the Maillard reaction.^(23)^ The fact that the DIAAS for English muffin and Texas toast were very low in this experiment is in agreement with data from previous experiments in which foods produced from cereal grains were used.^(17–20)^

The DIAAS obtained for hash brown was also less than the DIAAS (=100) that was calculated for potato protein,^(2)^ and it is possible that this reduction in DIAAS is also a consequence of the heat processing. However, although the first limiting AA in hash brown was lysine, the DIAA reference values for histidine, leucine, and the sulfur containing AA were also less than 100 for both age groups demonstrating that it was not only a low digestibility of lysine that resulted in a DIAAS value that was less than 100. According to FAO, a food can be considered an ‘excellent’ source of protein if the DIAAS is greater than or equal to 100, whereas foods with a DIAAS between 75 and 99 can be considered ‘good’ sources of protein.^(3)^ However, no claims for protein quality can be made if the DIAAS is less than 75.^(3)^ As a consequence, for individuals older than three years, hashbrowns can be considered a ‘good’ source of protein, whereas no claims for protein quality can be made for the breads regardless of age or for hash browns for children from 6 to 36 months old.

The DIAAS values of more than 100 in the cooked eggs for both age groups is in agreement with data for raw eggs.^(2)^ However, values in this study were greater than for raw egg, indicating that the cooking procedures used in this experiment may have improved AA digestibility, and therefore, increased DIAAS. This demonstrates that cooking with adequate temperatures and times can cause protein to be denatured without damaging its structure and enhance protein breakdown by proteolytic enzymes, consequently improving the digestibility and absorption of AA.^(29)^ It is also possible that cooking may have denatured avidin that may be bound to biotin and certain AA when eggs are in the raw form, which may have contributed to greater digestibility of AA, although the impact of avidin on AA digestibility is small.^(30)^ Therefore, SID values for AA of eggs calculated in this experiment were greater than what was previously reported for egg by-products.^(30)^ Nevertheless, the observation that the three cooked eggs had DIAAS values that were not different from each other implies that the type of cooking method did not influence protein quality. Therefore, all cooked eggs had ‘excellent’ protein quality for children from 6 to 36 months and for individuals older than 3 years.^(3)^

The observation that the first limiting AA for the combined meals of egg and English muffin or Texas toast was lysine, indicates that the inclusion of eggs in these meals at the inclusion rates used in this experiment was not sufficient to compensate for the low concentration of digestible lysine in the breads. Therefore, to obtain a DIAAS greater than 100, more eggs than what was used in this experiment need to be combined with the breads. Nevertheless, the two egg-bread combinations used had ‘good’ protein quality for both age groups demonstrating that combining eggs with English muffin or Texas toast increases the protein quality of the meal compared with consuming only the English muffin or the Texas toast. However, for the combined meal of scrambled egg and hash brown, there was no limiting AA, indicating that this combination provides all indispensable AA in sufficient quantities to complement each other, having an ‘excellent’ protein quality for both age groups.

Results of this experiment confirmed that values for SID are additive in combined meals as previously demonstrated,^(13)^ and as a result, prediction of DIAAS in combined meals from individual food ingredients is possible.^(22,26)^ Because people usually eat a combination of different food ingredients, this is an important feature of the DIAAS system, and if a nutrient database with CP and AA composition and corresponding SID values can be established, it is possible to predict DIAAS of different meals containing different combinations of food items to ensure that proteins complement each other and provide sufficient quantities of indispensable AA.

## Conclusions

Results demonstrated that cooked eggs are ‘excellent’ quality proteins for children from 6 to 36 months and individuals older than 3 years. No claims regarding protein quality can be made for English muffins or Texas toast. However, hash brown is a ‘good’ source of protein for individuals older than 3 years. The cooking method (boiling, frying, or scrambling) used to prepare the eggs did not influence protein quality. Because of the high DIAAS in eggs regardless of the type of preparation, cooked eggs complemented the plant-based proteins and provided meals with DIAAS > 75 or > 100 for both age groups, being considered ‘good’ or ‘excellent’ protein quality meals. However, for the egg-bread combinations, more eggs need to be consumed or another high-lysine food ingredient needs to be provided to obtain a meal that is adequate in all indispensable AA.

## Abbreviations

**AA:** Amino acids; **AID:** Apparent ileal digestibility; **CP:** Crude protein; **DIAA:** Digestible indispensable amino acid; **DIAAS:** Digestible indispensable amino acid score; **FAO:** Food and Agricultural Organization; **SE:** Standard error; **SID:** Standardised ileal digestibility.
